# The Physiological Effects of Ascending to Great Heights

**Published:** 1851-11

**Authors:** 


*The Physiological Effects of Ascending to great Heights.*—M. Payerne, of Cherbourgh, communicated observations which tended to prove that the feelings of lassitude and dyspnœa, which are experienced on ascending high mountains, are not attributable to an excess of oxygen in the air inspired as some physiologists have thought. M. Payerne has observed precisely the same effects produced under circumstances diametrically opposite; *i. e.* in descending under the water to the depth of forty-one metres (=44·838 yards.) M. Payerne stated that he had effected the descent by three different means, viz. by the ordinary diving-bell, by a diving bell that he had himself contrived, and in his sub-marine ship. In these several apparatus the auditory nerve had been differently affected. The first caused a disagreeable, almost painful, sensation during the period of submersion; the second produced similar effects only in descending or ascending; and the third, during the time requisite to establish the equilibrium with the medium in which he was thus placed. Under other points of view, the consequences were identical.

The author thus stated the results :—At thirty metres (=32·808 yds.) of depth of water, provided the temperature of the atmosphere respired did not exceed 10° Cent. (=50° Fahr.,) and at even less than this depth, with a higher temperature of the air, the workmen are obliged to rest from their work more frequently than when in the open air; and their arterial pulsations are notably increased. The descent and continuance under water do not give rise to hæmorrhages, but the returning to the surface in diving-bells, and the escape of the compressed air from the sub-marine boat on opening its doors to return to the ordinary atmosphere, have been known to be followed by epistaxis in some persons. In these cases, however, the hæmorrhage was not of the usual character, *i. e.* consisting of drops of blood of a more or less florid color, but it was a mere oozing of a saffron-colored fluid, thinner than blood,—a simple exudation, without rupture of the capilliaries. It could not be supposed that these effects were attributable to an insufficiency of oxygen, since a volume of air is condensed in proportion to the pressure to which it is subjected. At the greatest heights that have yet been reached, the air has still its relative proportion of oxygen.

M. Payerne added, that at small depths, *e. g.* a metre, a cubic metre of air will suffice for the respiration of four men, and it has even sufficed for five men, during one hour, and oxygen still remained.

The lassitude and dyspnœa which are experienced at great heights, are owing, according to M. Payerne, to a disturbance of the equilibrium between the tension of the fluids contained in our organs, and that of the circumambient atmosphere.—*Lon. Med. Gaz.*

